# Role of Multidetector Computed Tomography in Differentiation of Benign and Malignant Cavitary Lung Lesions With a Histopathological Correlation: A Retrospective Cross-Sectional Study

**DOI:** 10.7759/cureus.43005

**Published:** 2023-08-05

**Authors:** Navneet R Lal, Gaurav Raj Agarwal, Deb K Boruah

**Affiliations:** 1 Radiodiagnosis, Dr. Ram Manohar Lohia Institute of Medical Sciences, Lucknow, IND; 2 Radiodiagnosis, All India Institute of Medical Sciences, Guwahati, IND

**Keywords:** centrilobular nodules, malignant cavitary lesion, computed tomography (ct), wall thickness, cavitary lung lesion

## Abstract

Introduction

Cavitary lung disease has a wide range of differential diagnoses, which include both benign and malignant lesions. Imaging differentiation of benign from malignant cavitary lesions has always been a challenge due to overlapping imaging findings. The present study describes the most accurate multidetector computed tomography (MDCT) findings that could help in differentiating benign from malignant conditions in correlation with the histopathological reports.

Methods

This retrospective study was carried out on diagnosed cases of cavitary lung lesions on MDCT from January 2022 to February 2023. We evaluated the number of cavitary lung lesions, their location with respect to lung segment/lobe, the maximum diameter of the largest lesion, the maximum wall thickness of the largest cavity, and additional findings associated with these lesions. Measurements of the maximum wall thickness were plotted on a graph. Statistical analysis was done, and a receiver operating characteristic curve (ROC) was calculated to find the accurate cut-off wall thickness for malignant and non-malignant lesions. These findings were then correlated with the histopathological report.

Results

A review of the MDCT scans of 47 patients was done; 30 (63.8%) of those were male with a mean age of 47.93±14.68 (SD) years while 17 (36.2%) were female with a mean age of 52.53 ±18.38 (SD) years. Out of 47 patients, 27 (57.4%) had benign lesions and 20 (42.5%) had malignant lesions. Significant differences (p<0.05) were found between benign and malignant lesions while comparing the averages of maximum wall thickness (8.1 mm and 14.5 mm, respectively) and the irregular inner margin of the largest cavitary lesions. The presence of consolidation and centrilobular nodules correlated significantly (p<0.05) with the benign nature of cavitary lung lesions. The maximum cut-off wall thickness was <6 mm and >17 mm for the differentiation of benign from malignant lung lesions, respectively.

Conclusions

The maximum wall thickness and irregular inner margin of cavitary lung lesions was a good indicator for the differentiation of benign and malignant etiologies on MDCT while consolidation and centrilobular nodules favoured the benign etiology more.

## Introduction

A cavity is defined as a lucent gas-filled space surrounded by a wall located within pulmonary consolidation, a mass, or a nodule [1]. A cavitary lung lesion can be solitary or multiple. There is a wide list associated with the differential diagnosis of cavitary lung lesions, which can be categorized as benign or malignant in nature. The benign causes include tuberculosis, fungal infections, suppurative pneumonia, septic emboli, hydatid cyst, aspiration pneumonia, lung infarction, Wegener’s granulomatosis, etc. and the malignant causes can be primary lung mass or secondary metastasis [2]. So with such a wide differential list of cavitary lung lesions, it is important to differentiate between the benign and malignant etiologies [3]. Though X-ray and CT both are commonly used imaging modalities, CT plays a far better role in characterizing the cavitary lung lesion. CT gives a three-dimensional view of a cavitary lesion which helps in characterizing the wall thickness, margin, location, internal content, and other associated secondary findings [3].

The objective of this study was to find the most accurate imaging feature of cavitary lung lesions that can help in differentiating benign from malignant lung pathology.

## Materials and methods

Study population

This cross-sectional, retrospective study was done on obtained MDCT scans of 47 patients for a period of one year, from 10/01/2022 to 10/02/2023, in the tertiary hospital. The cases were searched using the terms like “cavitary lesion” and “mass with cavity” in the electronic reports and later cases were retrieved from the picture archiving and communication system (PACS) imaging system using the unique CT number of patients. The study included the patients in whom the chest MDCT showed one or more cavitary lesions. Patients without any etiological diagnosis were excluded from the study.

We obtained additional patient data like histopathological reports for correlation with CT findings from electronic medical reports. Definitive diagnoses of the cases were obtained through histopathological examination following a CT-guided biopsy, sputum smear microscopy, cartridge-based nucleic acid amplification test (CB-NAAT), and clinical/radiological follow-up. The diagnosis of TB was done based on the patient history, endemicity of the disease, imaging characteristics typical of TB, sputum microscopy, CB-NAAT, and bronchoalveolar lavage as per needs. Cases like pulmonary abscesses were diagnosed based on characteristic MDCT findings, patient history, improvement of the symptoms, and radiological findings on follow-up examination after empirical antibiotic treatment.

CT protocol

The images were obtained using 64-slice multidetector CT scanners (Philips Brilliance, Philips, Amsterdam, Netherlands) with the following parameters: tube voltage: 120 kVp; tube current: 250 mA; rotation time: 0.8 s; pitch: 1.375, section collimation: 0.625 mm, and reconstruction: 0.625 mm.

Core biopsy protocol

The location and accessibility of the lesion were assessed under CT guidance. All medical records were evaluated to declare the patient fit for the procedure. Prior to the intervention, the procedure was explained to the patient, and consent was obtained. Once the site of the biopsy was determined, a local anesthetic (2% lidocaine) was injected subcutaneously with a 25-gauge needle. A small surgical incision was made to facilitate easy passage of the needle through the skin. The core biopsy needle (semiautomatic biopsy needle 18 G, penetration depth 22 mm, needle length 10-16 cm) was introduced under CT guidance, and three to four samples were taken. The sample was kept in a 10% buffered formalin solution and sent for histopathological examination.

Image analysis

Images were analyzed by two thoracic radiologists with five years of experience at the same workstation (Philips). Disagreements between them were resolved by the senior radiologist with more than 10 years of experience in the same field, who gave the final diagnosis. The radiologists were kept blinded for clinical history and histopathological information of the patient. Evaluation of the CT scans of the 47 patients was done for the number of cavitary lesions, location based on the lung lobe, maximum wall thickness, largest cavity size, inner margin of the lesion, and associated secondary findings such as bronchiectasis, ground-glass opacity, centrilobular nodule, and consolidation.

High-resolution CT images were acquired in both pulmonary and mediastinal windows. Measurements of the cavitary lesions were done in the pulmonary window for better reproducibility of the result. Consolidation is defined as a homogeneous increase in lung parenchymal attenuation obscuring the margins of vessels and airway walls [1]. Ground-glass opacity is defined as hazy, increased opacity of the lung, with preservation of the bronchial and vascular margins. It is less opaque than consolidation, in which broncho-vascular markings are obscured [1]. Dot-like nodular opacity in the center of the normal secondary pulmonary lobule is considered centrilobular opacity. They are located within 1 cm of a pleural surface, indicating small-airways disease. The tree-in-bud pattern is one of the centrilobular abnormalities [1].

Statistical analysis

Statistical analysis was carried out with the SPSS program (Statistical Package for the Social Science version 16. SPSS Inc., Chicago). Mean ± SD, median, and IQR were used for data description. A p-value of < 0.05 was considered significant. For the comparison of rates, the chi-square test was used. The independent sample t-test and one-way analysis of variance (ANOVA) were used to compare the mean values. The optimal cut-off wall thickness to differentiate malignant lesions from benign ones was determined by receiver operating characteristic (ROC) curve analysis. Sensitivity and specificity were determined.

## Results

A total of 47 patients with at least one cavitary lesion on a CT chest were included in the study. The present study population included patients of all age groups, ranging from 15-80 years with a mean age of 49.60 ± 16.07 (SD) years (Table 1).

**Table 1:** Patient characteristics, lesion characteristics, etiological diagnosis, associated findings, and lobe involvement (n=47)

Patient characteristics	
Sex	Mean age
Female (n=17)	52.53±18.38
Male (n=30)	47.93±14.68
Lesion characteristics	
Wall thickness (mm)^a^	8.1±4.2
Malignant	14.5±6.5
Benign	10.8±6.1
Diameter (mm)	49±26
Malignant	48±23
Benign	49±28
Etiological diagnosis	
Benign pathologies (n=27)	
Pyogenic cavity	09 (34%)
Tuberculosis	06(22%),
Fungal infection/aspergillosis	04 (15%),
Lung abscess	03 (11%)
Aspiration with cavity	01 (4%)
Septic emboli	02 (7%)
Hydatid cyst	02 (7%)
Malignant pathologies (n=20)	
Primary lung mass	15 (75%)
Metastatic	05 (25%)
Associated findings (m=malignant, b=benign)	
Bronchiectasis (n=7)	m=4 (20%), b=3 (11%)
Consolidation ^a^ (n=16)	m=4 (20%), b=6 (22%)
Emphysema (n=6)	m=4 (20%), b=2 (7%)
Fibrosis (n=3)	m=1 (5%), b=2 (7%)
GGO (n=7)	m=5 (25%), b=2 (7%)
CLNO ^a^(n=8)	m=2 (10%), b=12 (46%)
Lung lobe involvement	
Right upper lobe (n=20)	m=07 (35%), b=13 (65%)
Right lower lobe (n=4)	m=02 (50%), b=02 (50%)
Left upper lobe (n=15)	m=09 (53%), b=07 (47%)
Left lower lobe (n=8)	m=02 (37%), b=05 (63%)
Nature of the lesion based on its inner margin ^a^	
Benign (n=27)	Irregular=8 (30%)
Malignant (n=20)	Irregular=16 (75%)
Data are presented as n ± standard deviation (SD) or n (%). a p<0.05 (malignant versus non-malignant).	

**Figure 1:**
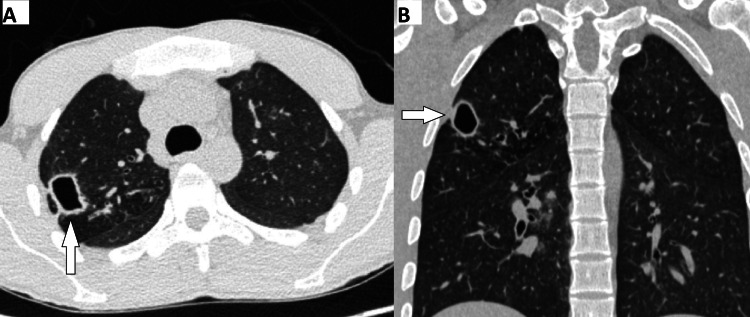
An 18-year-old male patient diagnosed with Mycobacterium tuberculosis following culture from sputum and bronchoalveolar lavage (A) Axial CT image shows a thin-walled cavitary lesion in the right upper lobe of the lung. (B) Coronal CT reformatted image demonstrates the smooth inner margin of the cavity with adjacent ground-glass opacity.

**Figure 2:**
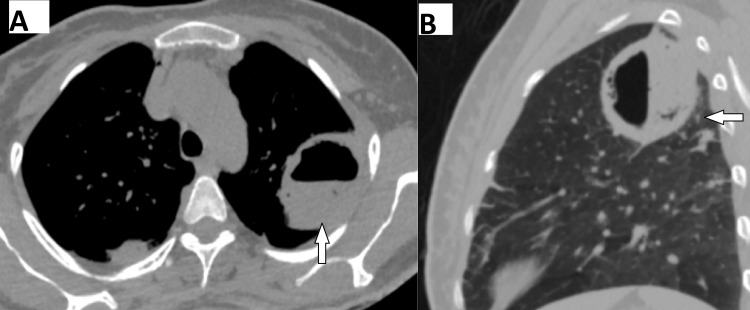
A 48-year-old female with a lung abscess (A) Axial and (B) Sagittal reformatted CT image reveals a thin-walled cavitary lesion with a regular inner margin in the left upper lobe of the lung with an air-fluid level and surrounding ground-glass opacity.

Of these, 30 (63.8%) were male with a mean age of 47.93 ± 14.68 (SD) years. Seventeen (36.2%) were female with a mean age of 52.53 ± 18.38 (SD) years. Benign and malignant lesions were diagnosed in 27 (57.4%) and 20 (42.55%) patients, respectively. Among the 27 patients with benign lesions, the diagnosis was a pyogenic cavity in nine (34%), tuberculosis (Figure 1) in six (22%), fungal cavity in four (15%), lung abscess (Figure 2) in three (11%), cavity following aspiration in one (4%), septic emboli with a cavity in two (7%), and hydatid cyst in two (7%).

**Figure 3:**
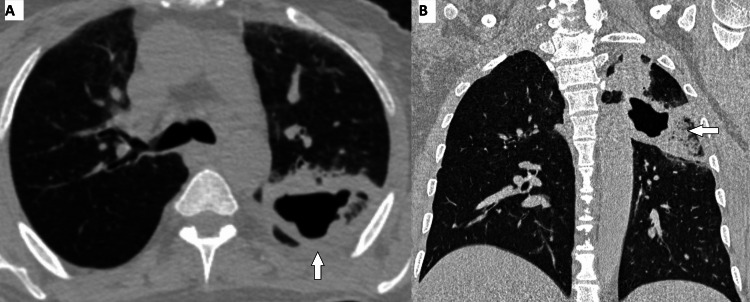
A 47-year-old man diagnosed with primary lung cancer following a CT-guided lung biopsy and histopathological examination (A) Axial CT image shows a thick-walled cavitary lesion with an irregular inner margin in the left upper lobe of the lung. (B) On a reformatted coronal CT image, we can see adjacent consolidation.

Out of 20 patients with malignant lesions, 15 (75%) were primary lung masses (Figure 3) and five (25%) were metastatic in nature.

The median diameter of the largest cavities was 45 mm (IQR, 27 - 62 mm), the median number of cavities observed was two (IQR, 1-3), the median thickness of the largest cavity was 9.6 mm (IQR, 5.8 - 14.6 mm).

The lesions were most commonly located in the right upper lobe followed by the left upper lobe. The most commonly associated findings with benign lesions were centrilobular nodules and consolidation. However, with malignant lesions, no significantly associated secondary findings were seen. Compared to females, males were more prone to both benign and malignant lesions. It was found that the mean age of the male population was lower. The irregular inner margin was more prevalent in malignant lesions, which correlated significantly (P<0.01). Similarly, regular inner margins were more prevalent in the benign lesions. The irregularity of the outer margin does not correlate significantly in differentiating benign from malignant lesions [4].

**Figure 4:**
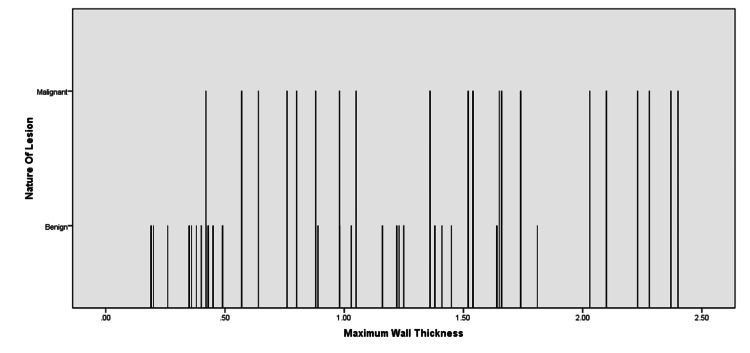
Histogram demonstrating the distribution of benign and malignant lesions based on wall thickness, where the malignant lesion number increases as the thickness of the cavity wall increases from left to right.

Figure 4 demonstrates the maximum wall thickness threshold for differentiating the benign from the malignant cavitary lesion derived from the ROC curve. Five mm (specificity 52% and sensitivity 95%) and 17 mm (specificity 97% and sensitivity 35%) were the best cut-off wall thicknesses for benign and malignant lesions, respectively (Table 2).

**Table 2:** Criterion cut-off values (~ 5 mm and ~ 17 mm) of the ROC curve for benign and malignant lesions, respectively ROC: receiver operating characteristic

Criterion (cut-off wall thickness value)	Sensitivity (%)	Specificity (%)
Malignant ≥ 17.4	35	97
Benign ≤ 5.3	95	52

**Figure 5:**
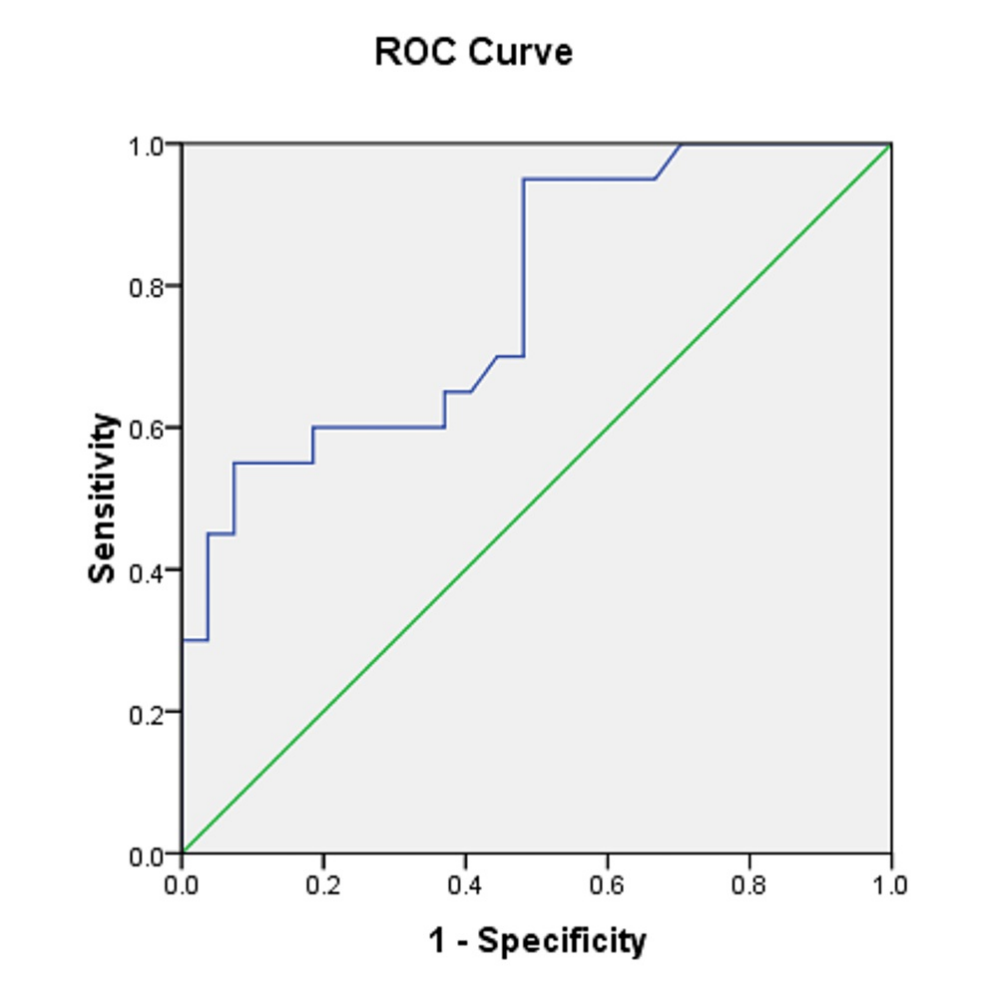
ROC curve for determining the maximum cut-off wall thickness for characterizing the benign and malignant cavitary lesions AUC = 0.783 ROC: receiver operating characteristic; AUC: area under the curve

Based on these maximum cut-off wall thickness values, it is seen that when the thickest part of the cavitary lesion is <5 mm, most of the lesions were benign (59%) as compared to malignant lesions (1%). When the thickest part of the lesion measured 5-17 mm, 37% of the lesions were benign and 45% were malignant. This indicated a percentage overlap between the benign and malignant lesions at 5-17 mm of wall thickness. However, if the greatest part of the wall thickness is kept >17 mm, 50% of the lesions were malignant compared to only 1% of benign lesions (Figure 5 and Table 3).

**Table 3:** The % distribution of benign and malignant lesions based on maximum wall thickness

	Maximum Wall Thickness			
Nature of lesion	<5 mm	5-17 mm	>17 mm	Total
Benign	16 (59%)	10 (37%)	1 (4%)	27
Malignant	1 (5.0%)	9 (45.0%)	10 (50.0%)	20
Total	15 (31.9%)	20 (42.5%)	12 (25.5%)	47

In the study, the averages of maximum wall thicknesses of benign and malignant cavitary lesions showed significant differences (8.1±4.2 mm vs 14.5±6.5 mm; p<0.05). The averages of the largest lesion diameter between the benign and malignant cavitary lesions were not significantly different (48.8±28.3 mm vs 48.6±22.8 mm; p>0.05).

## Discussion

Cavitary lesions, both benign and neoplastic, have a wide range of pathological distributions, and each lesion has a variable range of wall thickness from several millimeters to several centimeters [5,6]. In most of the studies, it was found that the most common causes of cavitary lung lesions were primary lung abscess, bronchogenic carcinoma, and tuberculosis, respectively, followed by metastatic tumors and fungal disease [7,8].

Some studies described that a combination of solitary cavities with thicker walls and irregular inner margins will favor the diagnosis of primary or metastatic lung cancer [7-10].

Several studies were done in the past to characterize the nature of lesions based on the wall thickness of cavitary lung lesions. In a study done by Woodring et al. based on chest X-rays, they found that a maximum wall thickness of 4 mm or less would be benign, and cavities with a maximum wall thickness of more than 15 mm would be malignant [11]. They found that 50% of the cavitary lesions in their study had wall thickness in the range of 5-15 mm. But in these cases, specific diagnoses from X-rays were not available, and a complete evaluation is necessary.

Nin et al., in the year 2016, plotted the ROC curve for the maximum wall thickness of cavitary lung lesions and found 24 mm and 7 mm as the cut-off wall thicknesses for characterizing malignant and benign cavitary lesions, respectively [12].

Though a number of different studies have been done previously characterizing the cavitary lung lesions as benign or malignant based on CT findings, most of these studies lack a definitive threshold for characterizing the lesion. And most of the time, these cases undergo guided biopsy or additional investigation delaying the early diagnosis and treatment of the disease. So it is the need of the hour to characterize these lesions based on CT findings to avoid unnecessary investigations into benign lesions, and additional investigations can be kept for more suspicious lesions.

In our study, we found that there was a significant difference in characterizing benign and malignant lesions based on wall thickness. A significant number of benign lesions (n=14) had a wall thickness of less than 5 mm and a significant number of malignant lesions (n=10) had a wall thickness of more than 17 mm. However, there was considerable overlap between the benign and malignant lesions at a wall thickness of 5-17 mm; similar findings were also stated in a previous study done by Woodring et al. [11]. The other findings that favored malignant over benign were the irregular inner margins of lesions.

In the present study, it was found that centrilobular nodular opacity and consolidation were more frequently associated with benign cavitary lesions. This finding was in accordance with the previous study done by Nin et al. [12], which states that the centrilobular nodular opacity was commonly found in patients with mycobacterial infection, pyogenic infection, and fungal or viral infection [13-15] as compared to malignant etiology.

So from the result of the present study, it is seen that the maximum wall thickness is the most reliable criterion for differentiating benign and malignant cavitary lung lesions. And the most accurate cut-off wall thicknesses were 5 mm and 17 mm for differentiating benign and malignant cavitary lung lesions, respectively. These cut-off wall thicknesses would avoid unnecessary interventions in benign lesions, which could be reserved for suspicious malignant lesions, thus avoiding the loss of precious time for early planning in the management of patients.

The limitations to be considered in our study were the small sample size and the retrospective nature of the study, creating problems in data collection.

## Conclusions

In conclusion, we found that CT is a valuable and emerging tool in characterizing benign and malignant cavitary lung lesions. We demonstrated two characteristic CT findings that can be used as an etiological tool for characterizing cavitary lung lesions. The maximum wall thickness and irregular inner margin of the lesion favored a malignant nature. The associated additional findings like centrilobular nodular opacity and consolidation favored benign lesions. However, the size of the cavity and its location with respect to the lobe were not capable of differentiating benign and malignant cavitary lesions.
